# Understanding Problems With Sleep, Sexual Functioning, Energy, and Appetite Among Patients Who Access Transdiagnostic Internet-Delivered Cognitive Behavioral Therapy for Anxiety and Depression: Qualitative Exploratory Study

**DOI:** 10.2196/15037

**Published:** 2020-10-13

**Authors:** Michael R Edmonds, Heather D Hadjistavropoulos, Kirsten M Gullickson, Aleiia JN Asmundson, Blake F Dear, Nickolai Titov

**Affiliations:** 1 Online Therapy Unit Department of Psychology University of Regina Regina, SK Canada; 2 eCentreClinic Department of Psychology Macquarie University Sydney Australia

**Keywords:** cognitive behavioral therapy, anxiety, depression, internet-based intervention, sleep, sexual health, motivation, appetite

## Abstract

**Background:**

Transdiagnostic internet-delivered cognitive behavioral therapy (T-ICBT) is an effective treatment for anxiety and depression, and nowadays, there is interest in exploring ways to optimize T-ICBT in routine care. T-ICBT programs are designed to address the primary cognitive-affective and behavioral symptoms of anxiety and depression (eg, low mood, worry, anhedonia, and avoidance). Treatment also has the potential to resolve other symptom concerns (eg, sleep disruption, sexual dysfunction, lack of energy, and appetite or weight changes). Having additional information regarding the extent of these concerns and how concerns change over time could prove beneficial for further development of T-ICBT in routine care.

**Objective:**

This exploratory formative study aims to better understand sleep, sexual functioning, energy, and appetite concerns among T-ICBT clients seeking treatment for depression and anxiety. A qualitative analytic approach was used to identify themes in the symptom concerns reported by patients in the areas of sleep, sexual functioning, energy, and appetite at the time of enrollment. Patient responses to related items from screening measures for anxiety and depression were also examined pre- and posttreatment.

**Methods:**

Patients in routine care who applied for a T-ICBT program for depression and anxiety over a 1-year period were included in this study. As part of the application and screening process, participants completed depression and anxiety symptom measures (ie, 9-item Patient Health Questionnaire and 7-item Generalized Anxiety Disorder scale). These same measures were administered posttreatment. Subsequently, they were asked if they were experiencing any problems with sleep, sexual activity, energy, or appetite (yes or no). If their response was yes, they were presented with an open-ended comment box that asked them to describe the problems they had experienced in those areas.

**Results:**

A total of 462 patients were admitted to T-ICBT during the study period, of which 438 endorsed having some problems with sleep, sexual activity, energy, or appetite. The analysis of open-ended responses indicated that 73.4% (339/462) of patients reported sleep problems (eg, difficulty initiating or maintaining sleep), 69.3% (320/462) of patients reported problems with energy or motivation (eg, tiredness and low motivation), 57.4% (265/462) of patients reported appetite or body weight concerns (eg, changes in appetite and weight loss or gain), and 30.1% (139/462) of patients described concerns with sexual functioning (eg, loss of interest in sex and difficulty with arousal). Item analysis of symptom measures demonstrated that T-ICBT produced improvements in sleep, energy, and appetite in 8 weeks. Sexual dysfunction and weight changes were not represented in the screening measures, so it remains unclear what effect T-ICBT has on these symptoms.

**Conclusions:**

Sleep disruption, lack of energy, appetite or weight changes, and sexual dysfunction are common concerns reported by clients enrolled in T-ICBT in routine practice and may deserve greater attention in T-ICBT program development and administration.

## Introduction

### Background

Anxiety and depression are prevalent, disabling, and frequently comorbid mental health conditions that are associated with a range of negative outcomes, including a number of physical health problems, lower marital satisfaction, absenteeism or presenteeism in the workplace, and financial difficulties [1]. Transdiagnostic internet-delivered cognitive behavioral therapy (T-ICBT) has emerged as an efficient and effective treatment for individuals who experience concurrent symptoms of anxiety and depression [2]. T-ICBT generally involves clients completing weekly web-based lessons and homework that teach cognitive and behavioral techniques for managing symptoms [3]. The transdiagnostic approach is based on the premise that anxiety and depression are often comorbid and share common symptoms, such as maladaptive thinking patterns and avoidance [4,5]. T-ICBT protocols typically teach clients a variety of symptom management strategies that are applicable to both anxiety and depression, including cognitive restructuring, behavioral activation, and graded exposure [6]. Although some T-ICBT programs are unguided (ie, the patient completes the program independently), many are therapist assisted (ie, the patient has regular contact with a web-based therapist). In therapist-assisted T-ICBT programs, support is typically provided through secure messaging or over the phone wherein therapists answer patient questions, offer guidance and encouragement, and provide feedback on symptom changes [3].

Considerable evidence supports the efficacy of T-ICBT, and as a result, it is increasingly being implemented in routine care [7]. T-ICBT protocols are particularly appealing because they streamline the treatment process for patients and therapists [2,4]. From the patient’s perspective, T-ICBT produces equally impressive treatment effects as disorder-specific internet-delivered cognitive behavioral therapy (ICBT) while saving patients from the burden of having to complete several treatment protocols sequentially to address comorbid conditions [2,4,8-11]. From the therapist’s perspective, T-ICBT protocols simplify referral decision making and training for staff who can learn and implement one transdiagnostic program rather than several disorder-specific programs [2,4]. Although T-ICBT programs show considerable promise, it remains a relatively new form of treatment in routine care; thus, it is important to learn from patients and continually seek to improve treatment to meet the needs of patients [12].

T-ICBT programs are designed to address the primary cognitive-affective and behavioral symptoms of anxiety and depression (eg, low mood, worry, anhedonia, and avoidance), yet successful treatment has the potential to resolve many additional symptom concerns that are present with anxiety and depression. For example, internalizing disorders such as anxiety and depression share common features, such as sleep problems, sexual dysfunction, loss of energy, and changes in appetite/weight [13-20]; however, little is known about T-ICBT clients’ concerns in these areas at the beginning of T-ICBT and the impact of T-ICBT on these additional concerns. Formative research that seeks to improve understanding of diverse symptoms pre- and posttreatment could be beneficial for identifying opportunities to improve the delivery of T-ICBT in routine care.

### Objectives

The goal of this exploratory study is two-fold. First, we aim to better understand the nature of client concerns about sleep, sexual functioning, energy, and appetite/weight at the time of enrollment by analyzing responses to an open-ended question about problems with sleep, sexual functioning, energy, and appetite or weight at pretreatment. Qualitative research is an excellent method for examining patient concerns [21]. Second, following an approach similar to qualitative assurance studies [22], item-level data from screening measures of anxiety and depression were examined to explore whether T-ICBT was associated with symptom improvements in these areas at posttreatment. Given the nature of depression and anxiety, we hypothesized that clients would commonly report experiencing problems with sleep, sexual functioning, energy, or appetite/weight. In terms of the nature of the clients’ concerns, we expected clients to report sleep problems such as insomnia or hypersomnia, sexual problems such as reduced sexual desire and difficulties with arousal, fatigue or tiredness, and changes in appetite and accompanying weight change. We also hypothesized that item analysis would show improvements in these areas posttreatment; however, we did not have hypotheses about the magnitude of symptom reduction.

## Methods

### Study Design and Ethics

This study involved extracting patient records from the Online Therapy Unit, which is a government-funded web-based therapy clinic at the University of Regina that freely offers ICBT on a routine basis to residents of Saskatchewan. In addition to providing T-ICBT, the Online Therapy Unit conducts research to optimize the delivery of ICBT in routine care; thus, patients who receive T-ICBT are asked for consent to use their deidentified data for research purposes. This study involved retrieving records from 462 patients who received T-ICBT with once-a-week therapist support over a 1-year period (June 1, 2016, to May 31, 2017). We did not retrieve additional data from patients who received ICBT for chronic pain or received T-ICBT without regular weekly therapist support. Of note, 245 of the 462 patient records were part of a registered trial (ISRCTN14230906) [23], but as they received standard T-ICBT with the same once-a-week therapist support, their data were included in this study.

### Participants and Procedure

Individuals who participated in this study reported that they heard about the Online Therapy Unit through a variety of sources, including referral from a community mental health clinic (170/462, 36.5%), referral by their family physician (101/462, 21.7%), word of mouth (69/462, 14.8%), media (57/462, 12.2%), searches on the web and email announcements (55/462, 11.8%), or printed posters or cards (14/462, 3%). Individuals who wished to take part in the Wellbeing Course began by completing a brief web-based screening questionnaire. The questionnaire ensured that participants met the basic inclusion criteria, including (1) being aged 18 years or older; (2) residing in Saskatchewan, Canada; (3) endorsing symptoms of depression and/or anxiety; (4) being comfortable using a computer with access to the internet; and (5) willing to consent to treatment and provide a medical contact for emergency purposes. Of note, a formal diagnosis of an anxiety or mood disorder was not required to participate in the program, but all patients endorsed at least some symptoms of anxiety or depression.

Those who met the basic inclusion criteria then completed additional web-based screening questions about their background (eg, demographic characteristics and contact information) and symptoms (eg, anxiety and depression). Specifically, patients completed the 7-item Generalized Anxiety Disorder (GAD-7) scale and the 9-item Patient Health Questionnaire (PHQ-9), widely used screening measures that ask patients to report the frequency of anxiety and depression symptoms over the previous 2 weeks [24,25]. The PHQ-9 and GAD-7 are formatted and scaled very similarly, with response options ranging from 0 (*not at all*) to 3 (*nearly every day*), resulting in possible scores between 0 and 21 for the GAD-7 and between 0 and 27 for the PHQ-9. Of relevance to this study were items 3, 4, and 5 from the PHQ-9, which assessed symptoms of sleep, energy, and weight. Participants with scores greater than 5 on the GAD-7 or PHQ-9 were considered eligible for T-ICBT. An additional interest to this study, participants were asked to answer the yes or no question: “Have you had any problems with sleep, appetite/weight, energy level, or sexual activity?” following the symptom measures. If they responded “yes,” participants were presented with an open-ended comment box that asked them to provide details about the difficulties they had experienced in those areas.

After completion of the web-based screening questionnaire, participants were briefly interviewed by a clinician via the telephone to further assess suitability for the Wellbeing Course. Specifically, telephone screeners would provide more information about the nature of the Wellbeing Course to ensure clients were interested and ask any additional questions to determine whether patients should be excluded because they (1) were considered high risk for suicide or reported very severe symptoms (n*=*55), (2) were seeking primary treatment for another disorder (ie, obsessive compulsive disorder, posttraumatic stress disorder, bipolar disorder, psychotic symptoms, and alcohol or drug problems; n=21), (3) were receiving regular concurrent in-person psychotherapy (eg, receiving face-to-face cognitive behavioral therapy [CBT] in an outpatient setting) that is expected to address the client’s concern (n=6), or (4) did not meet the inclusion criteria (eg, age or lack of computer access or skills) described earlier (n=21).

All patients accepted for treatment received the same T-ICBT intervention, a program called the Wellbeing Course, which was developed by the eCentreClinic in Sydney, Australia. The Wellbeing Course has previously been studied in numerous clinical trials and found to be effective in reducing symptoms of anxiety and depression [4,6,8,9,26], but specific examination of how T-ICBT impacts sleep, sexual functioning, energy, and appetite/weight has not been undertaken. The Wellbeing Course is 8 weeks in duration and comprises 5 core lessons designed to target symptoms of depression and/or anxiety: (1) the cognitive behavioral model, (2) thought monitoring and challenging, (3) dearousal strategies and pleasant activity scheduling, (4) gradual exposure, and (5) relapse prevention. Each lesson consists of psychoeducation, instruction on cognitive behavioral strategies used for symptom reduction, case examples, and homework exercises. Participants also have access to additional resources on topics that are relevant to depression and anxiety (ie, assertiveness, communication, problem solving, worry time, and sleep). As they work through the course, patients receive therapist assistance in the form of weekly emails and occasional telephone calls. Patients complete the PHQ-9 and GAD-7 symptom measures again after completing the 8-week course.

### Data Analysis

Qualitative content analysis was used to explore patients’ responses to the open-ended question about their problems with sleep, sexual functioning, energy, and appetite/weight [27]. A conventional or inductive approach was used, wherein the data were coded without the use of a preexisting coding guide [27]. The researchers involved with data analysis had all received didactic training in qualitative research methods, and two researchers had experience publishing qualitative research.

Data analysis was an iterative process that began with one undergraduate research assistant (AA) reading each response closely to obtain an initial impression of the data and engage in open coding, wherein basic codes representing each unit of meaning were derived (eg, difficulty falling asleep). Subsequently, the research assistant met with a PhD student in psychology (ME) to discuss initial impressions and create a preliminary coding guide of keywords and definitions. The research assistant then comprehensively coded all participant responses using the new coding guide. Next, several researchers (AA, ME, and HH) came together to sort the individual codes into meaningful themes and developed definitions for each theme (eg, the theme *difficulty falling asleep* was defined to capture any client statement that describes problems with initiating sleep). Finally, to ensure the accuracy of the coding, all participant responses were recoded by a second PhD student in psychology (KG) using the finalized coding guide. The lead author resolved a small number of coding discrepancies between the first and second coders and completed a final review of the data. After coding was complete, the identified themes (Table 1) were analyzed using descriptive statistics to determine which concerns and symptoms were reported most frequently by participants.

In addition, to preliminarily explore the impact of T-ICBT on sleep, sexual functioning, energy, and appetite/weight from pre- to posttreatment, an item analysis was conducted on patients’ responses on the GAD-7 and PHQ-9. Specifically, descriptive statistics were used to calculate the percentage of clients endorsing each item pretreatment as well as mean symptom change scores from pre- to posttreatment. Although we included all items in the analysis, we were particularly interested in the items related to sleep (PHQ-9 item 3: “trouble falling or staying asleep, or sleeping too much”), energy (PHQ-9 item 4: “feeling tired or having little energy”), and appetite (PHQ-9 item 5: “poor appetite or overeating”). Neither the GAD-7 nor the PHQ-9 included an item to assess weight or sexual functioning; thus, we were unable to determine if T-ICBT helped resolve these problems.

**Table 1:** Description of each theme along with subcategories, representative quotes, and frequencies (N=462).

Theme and description	Subcategories	Representative quotes	Frequency, n (%)
Sleep: concerns about quantity or quality of sleep	Difficulty falling asleep Difficulty staying asleep Difficulty returning to sleep Early waking Difficulty waking up Sleeping too much Nightmares General sleep problems	“I have troubles falling asleep, staying asleep, and when I finally fall asleep it is time to wake up and I have trouble waking up.” “Most days [I] wake up feeling unrested, even if I got to bed at a decent time.” “[I] want to sleep all the time.”	339 (73.4)
Energy or motivation: concerns about lack of energy, motivation, and self-care	Decrease in energy Feeling tired or exhausted Mentally tired Lacking motivation Little or no exercise	“I feel tired all the time. [...] I can’t even muster energy to exercise or do things I used to like doing.” “I don’t want to do anything except sleep or sit.” “I’m just very very tired, exhausted, low energy, and have no motivation.”	320 (69.3)
Appetite/weight: concerns related to eating, appetite, and weight changes	Variable appetite Appetite greater than usual Appetite less than usual Poor diet Emotional eating Weight loss Weight gain Weight fluctuations Overweight Unsuccessful weight loss Unsuccessful weight gain	“Some days my appetite is very poor, some days I feel like eating all the time.” “I have no appetite and usually just eat because I have to.” “In the last year I have gained 25lbs due to depression and not being able to continue with my normal active lifestyle.”	265 (57.4)
Sexual functioning: concerns with low sexual interest or engagement	Low sex drive Difficulty with arousal Lack of sexual activity	“My sex drive is pretty much non-existent.” “Sexually, [I am] unable to get aroused.” “I have been avoiding intimacy.”	139 (30.1)
Cognitive-affective symptoms: concerns related to emotion regulation or cognitive functioning	Anxiety Depression Angry and irritable Low self-esteem Emotional fluctuations Lack of focus Inability to relax Cannot turn off mind Constant worry Overthinking	“[I] cry regularly. [I am] irritable and moody.” “I lose concentration easily and feel foggy.” “[I] can’t relax.”	117 (25.3)
Somatic symptoms: concerns about physical symptoms and overall health	Digestive problems Nausea Dizziness General health concerns	“I constantly feel sick [to my stomach].” “[In] the evening I feel pressure behind my eyes and a slight headache. “ “[I am] always sick with colds and flus.”	30 (6.5)

## Results

### Sample Characteristics

A total of 462 patients enrolled in the Wellbeing Course during the study period (see Table 2 for sample characteristics) and completed initial screening measures. Of the 442 patients who began treatment by logging in to the website, 341 (77.1%) completed 4 of the 5 core lessons and 298 (67.4%) completed all 5 lessons and completed posttreatment measures. Almost three-fourths of the participants were female (339/462, 73.4%), and the average age was 37 years (SD 12.33). The majority of participants reported being in a relationship and living in a city with more than 10,000 people. Participants varied widely in education level, although only 2.8% (13/462) had less than a high school education. Most participants reported being employed (283/462, 61.3%).

**Table 2:** Participant characteristics (N=462).

Variables		Values
**Age (years)**		
	Mean (SD)	37.12 (12.33)
	Range	18-86
**Gender, n (%)**		
	Male	119 (25.8)
	Female	339 (73.4)
	Other	4 (0.9)
**Marital status, n (%)**		
	Single or never married	104 (22.5)
	Married	226 (48.9)
	Living with partner	81 (17.5)
	Separated or divorced	45 (9.7)
	Widowed	6 (1.3)
**Education, n (%)**		
	Less than high school	13 (2.8)
	High school diploma	82 (17.7)
	College certificate or diploma	135 (29.2)
	Some university	64 (13.9)
	University undergraduate degree	110 (23.8)
	University professional or graduate degree	58 (12.5)
**Employment status, n (%)**		
	Employed full time	224 (48.5)
	Employed part time	59 (12.8)
	Unemployed	38 (8.2)
	Homemaker or retired or student	97 (21.0)
	Short- or long-term disability	44 (9.5)
**Location, n (%)**		
	Large city (>200,000)	185 (40.0)
	Small to medium city (10,000-200,000)	153 (33.1)
	Town or village	94 (20.3)
	Farm	29 (6.3)
	Reserve	1 (0.2)

### Problems With Sleep, Sexual Functioning, Energy, and Appetite

The vast majority of patients (438/462, 94.8%) responded “yes” to the question asking if they had experienced problems related to sleep, sexual functioning, energy, or appetite/weight and thus responded to the open-ended comment box asking them to provide more detail about their problems in these areas. Patients described problems with sleep, sexual functioning, energy or motivation, and appetite/weight; however, they also reported on cognitive-affective symptoms and somatic symptoms. Table 1 includes descriptions, examples, representative quotes, and frequencies for each identified category. The most commonly reported problems were sleep, energy/motivation, and appetite/weight. Of the patients who responded to the open-ended comment box, 13.7% (60/438) provided a response that was coded into only 1 category, 24.8% (109/438) of responses were coded into 2 categories, 37.7% (165/438) were coded into 3 categories, 18.3% (80/438) were coded into 4 categories, 4.8% (21/438) were coded into 5 categories, and 0.9% (4/438) were coded into all 6 categories.

### Treatment Outcomes and Item Analysis

Overall, patients’ anxiety and depression scores decreased over the course of treatment. The mean total scores at the time of enrollment were 12.6 (SD 5.0) on the GAD-7 and 12.5 (SD 6.1) on the PHQ-9, which represents moderate symptoms of anxiety and depression. In this sample, treatment resulted in a mean score reduction at posttreatment of 6.6 points (SD 5.1) on the GAD-7 and 5.9 points (SD 5.1) on the PHQ-9, indicating that patients’ anxiety and depression symptoms were in the mild range at the end of treatment. The results from the GAD-7 and PHQ-9 item analyses are presented in Table 3. Of the symptoms of interest in this study, low energy was the most frequently endorsed item on the PHQ-9, with 94.1% (428/455) of patients indicating they had felt tired or had little energy several days or more over the last 2 weeks. Sleep disruption was the second most commonly endorsed symptom, with 87.9% (400/455) of patients indicating they experienced trouble falling or staying asleep or sleeping too much on several days or more over the last 2 weeks. Appetite problems were another commonly endorsed item on the PHQ-9, with 79.3% (361/455) of patients indicating they experienced poor appetite or overeating several days or more over the last 2 weeks. Promisingly, T-ICBT appeared to have a positive impact on sleep, energy, and appetite symptoms, that is, patients reported a decrease in the frequency of low energy, sleep disturbance, and appetite problems from pre- to posttreatment. It is important to note, however, that patients still reported experiencing symptoms in these areas posttreatment. The lack of GAD-7 or PHQ-9 items related to sexual functioning and weight meant it was not possible to assess whether T-ICBT led to improved functioning in those areas.

**Table 3:** Frequencies of responses to the 7-item Generalized Anxiety Disorder scale and the 9-item Patient Health Questionnaire items at pretreatment (N=462).

Item and symptom		Distribution of pretreatment responses					Pretreatment mean score (SD)		Posttreatment mean score (SD)
		0 (not at all), n (%)	1 (several days), n (%)	2 (more than half the days), n (%)	3 (nearly every day), n (%)				
**7-item Generalized Anxiety Disorder (n=454)**									
	Feeling nervous or anxious	12 (2.6)	127 (28.0)	143 (31.5)	172 (37.9)	2.05 (0.87)		0.99 (0.80)	
	Uncontrollable worrying	27 (5.9)	125 (27.5)	129 (28.4)	173 (38.1)	1.99 (0.95)		0.83 (0.88)	
	Worrying about a variety of things	13 (2.9)	114 (25.1)	137 (30.2)	190 (41.9)	2.11 (0.88)		0.95 (0.89)	
	Trouble relaxing	40 (8.8)	130 (28.6)	130 (28.6)	154 (33.9)	1.88 (0.98)		0.81 (0.90)	
	Restlessness	145 (31.9)	162 (35.7)	91 (20.0)	56 (12.3)	1.13 (1.00)		0.52 (0.80)	
	Irritability	24 (5.3)	142 (31.3)	128 (28.2)	160 (35.2)	1.93 (0.94)		1.02 (0.97)	
	Fearing something awful	96 (21.1)	144 (31.7)	109 (24.0)	105 (23.1)	1.49 (1.07)		0.60 (0.86)	
**9-item Patient Health Questionnaire (n=455)**									
	Anhedonia	54 (11.9)	201 (44.2)	107 (23.5)	93 (20.4)	1.53 (0.95)		0.76 (0.80)	
	Feeling depressed and hopeless	51 (11.2)	203 (44.6)	117 (25.7)	84 (18.5)	1.51 (0.92)		0.76 (0.79)	
	Sleep disruption	55 (12.1)	122 (26.8)	118 (25.9)	160 (35.2)	1.84 (1.04)		1.00 (0.93)	
	Low energy	27 (5.9)	127 (27.9)	128 (28.1)	173 (38.0)	1.98 (0.95)		1.12 (0.90)	
	Poor appetite or overeating	94 (20.7)	133 (29.2)	111 (24.4)	117 (25.7)	1.55 (1.09)		0.81 (0.93)	
	Feeling bad about self	59 (13.0)	163 (35.8)	122 (26.8)	111 (24.4)	1.63 (0.99)		0.69 (0.85)	
	Trouble concentrating	100 (22.0)	147 (32.3)	117 (25.7)	91 (20.0)	1.44 (1.04)		0.62 (0.83)	
	Psychomotor agitation or retardation	236 (51.9)	124 (27.3)	67 (14.7)	28 (6.2)	0.75 (0.92)		0.31 (0.60)	
	Self-harm or suicidality	359 (78.9)	69 (15.2)	19 (4.2)	8 (1.8)	0.29 (0.63)		0.11 (0.38)	

## Discussion

### Principal Findings

This formative study qualitatively examined patients’ self-reported concerns with sleep, sexual functioning, energy, and appetite/weight at the time of enrollment in T-ICBT for depression and anxiety to learn more about the prevalence and nature of patients’ problems in these areas. In addition, an item analysis was conducted on screening measures of anxiety and depression to explore whether T-ICBT produces improvements in these symptoms posttreatment. Of importance, the vast majority of T-ICBT clients (438/462, 94.8%) endorsed problems in at least one area and 87.4% (404/462) of clients reported problems in more than one area. Although this is not surprising given research has demonstrated that sleep problems, sexual dysfunction, lack of energy, and appetite/weight changes are shared features of anxiety and depression [13-20], the extent of the problems is important to understand so as to inform T-ICBT delivery in routine care.

Qualitative analysis of patients’ descriptions of their concerns revealed that sleep disturbance was the most common problem described by clients (339/462, 73.4%). As hypothesized, clients’ sleep concerns were consistent with insomnia (eg, difficulty falling asleep, difficulty staying asleep, and early waking) and hypersomnia (eg, difficulty waking up, and sleeping too much), although a number of clients also reported having nightmares. Loss of energy or motivation was the second most common client concern (320/462, 69.3%). Consistent with our expectations, clients described experiencing a loss of energy and mental or physical tiredness; however, they also reported a lack of motivation and loss of interest in self-care. Interestingly, clients often discussed energy and motivation in tandem, which is why these problems are grouped together in the results. The third most common client concern was changes in appetite/weight (265/462, 57.4%), with some clients describing an increase in appetite/weight and others a decrease in appetite/weight. This is mostly consistent with our hypothesis, but we did not fully anticipate how widespread problems would be with poor diet or emotional eating. A less common concern, although still affecting one-third of the sample, was sexual dysfunction (139/462, 30.1%). In this domain, clients reported loss of sexual desire and difficulty with arousal, which is in line with our hypothesis.

Although the question presented to clients asked specifically about sleep, sexual functioning, energy, and appetite/weight, clients also described problems in other areas without being prompted to do so. Specifically, 25.3% (117/462) of clients reported experiencing cognitive-affective symptoms (eg, worry, low mood, anger or irritability, lack of focus, and inability to relax), and 6.5% (30/462) of clients described somatic symptoms (eg, digestive problems, nausea, dizziness, and compromised immune system). These concerns were not the intended focus of this study, yet the fact that they were brought up by clients suggests they are important. It remains to be seen if these concerns would be more prevalent if the question prompted clients to think about these domains.

The results of the item analysis provided preliminary evidence that T-ICBT leads to improvements in sleep, energy, and appetite in 8 weeks. This promising finding suggests that T-ICBT has a positive impact on problems in these areas. It is important to note, however, that patients’ problems with sleep, energy, and appetite were not eliminated entirely, which suggests that it may still be possible to optimize T-ICBT to better address these problems. Moreover, we were unable to monitor changes in sexual functioning or weight over the course of treatment because these symptoms were not represented by the items on the GAD-7 of PHQ-9. This seems especially problematic for sexual functioning, an issue that was raised by more than one-third of the study sample. A recent study found that face-to-face CBT resulted in modest reductions in reports of sexual dysfunction [20], but it is unknown if T-ICBT produces similar reductions in sexual concerns.

The findings of this study have implications for future T-ICBT research with regard to symptom screening and outcome monitoring. Specifically, single-item measures can provide some indication of symptom change over the course of treatment, but it is unclear whether they are sensitive enough to the wide variety of problems patients reported with regard to sleep, energy, appetite/weight, and sexual functioning. Thus, there may be value in expanding screening or outcome measures to include more through assessment of sleep, sexual functioning, energy or motivation, and appetite/weight. Enhancing existing screening or outcome measures would allow researchers to make concrete conclusions about the efficacy of T-ICBT in improving these symptoms. However, it would be important to balance the desire for more detailed information with the increased burden on clients when completing screening and posttreatment questionnaires. If measures were to be added, they would need to be brief, psychometrically sound, and not redundant with existing items or measures. In the long term, learning about the effects of T-ICBT on sleep, sexual functioning, energy or motivation, and appetite/weight might provide insight into ways to enhance treatment content, which, in turn, might influence treatment engagement or outcomes. The results of this study also have implications for clinical practice and future T-ICBT program development. With regard to therapist support, the results of this study emphasize that therapists need to be aware of how common and diverse problems with sleep, energy or motivation, appetite/weight, and sexual functioning are within their client group and provide support accordingly. With respect to program development, the results of this study suggest that concerns related to sleep, energy or motivation, appetite/weight, and sexual functioning are common, and ensuring program content relates to these concerns (eg, by using symptoms of sleep disruption in case examples provided to patients) could be an important part of maximizing the patient acceptability of T-ICBT.

### Strengths and Limitations

This study makes a valuable contribution to the literature because it is the first to qualitatively examine the prevalence and nature of T-ICBT clients’ self-described problems with sleep, sexual functioning, energy, and appetite/weight. The qualitative approach to data analysis provided a depth of information unavailable in previous quantitative studies. For example, we found that clients’ specific concerns about sleep, sexual functioning, energy or motivation, and appetite/weight are diverse and may not be adequately captured by common symptom screening measures. The inclusion of a large sample of clients increases our confidence that the results are generalizable to other clients seeking T-ICBT in routine care. The addition of the item analysis is another notable strength of the study, as it provides preliminary evidence that T-ICBT has some positive effects on sleep disruption, low energy, and appetite change.

This study also had several limitations. First, the wording of the question patients responded to may have prompted clients to respond in a certain way. Patients were specifically asked only a single question about sleep, sexual functioning, energy, and appetite/weight, and using a more open-ended question or asking about each symptom domain separately may have produced different results. Second, in terms of question order, clients responded to the research questions after they had completed the GAD-7 and PHQ-9, which makes it possible that clients were primed by the items related to sleep, energy, appetite, and cognitive-affective symptoms. In interpreting the results, readers should also consider that the PHQ-9 and GAD-7 measure the frequency of symptoms but not the severity of the symptoms, and there is no way to know from our data what proportion of clients were experiencing clinically significant difficulties in each area. It is also important to note that the characteristics of the sample used in this study may limit the generalizability of the results to other ICBT programs. The characteristics of the sample that should be considered by the reader when interpreting the results include the fact that a large proportion of patients were self-referred to the service (193/462, 41.8%), that 58.9% (272/462) of patients reported receiving simultaneous pharmacological treatment for their concerns at pretreatment, and that patients who were receiving simultaneous in-person therapy (n=6) were excluded from the study. Posttreatment results must also be interpreted with caution, as 32.6% (144/442) of participants had withdrawn from treatment before the 8-week point, and the extent to which patients used skills described in the course or gained knowledge was not assessed in this study.

### Conclusions

This study presents an overview of the concerns related to sleep, energy, appetite, and sexual functioning reported by individuals seeking T-ICBT for anxiety and depression and may therefore be of interest to program designers and clinicians interested in offering T-ICBT. We found that the majority of clients reported symptoms such as sleep disruption, lack of energy or motivation, and appetite/weight changes, and just less than one-third of the sample reported experiencing sexual dysfunction. We also found preliminary evidence that T-ICBT resulted in reductions in the reported frequency of sleep disruption, low energy, and appetite disturbance over 8 weeks. The results of this study allowed us to identify several areas where T-ICBT outcome measures could be improved and provided a number of directions for future research.

## Abbreviations

CBT: cognitive behavioral therapy
GAD-7: 7-item Generalized Anxiety Disorder
ICBT: internet-delivered cognitive behavioral therapy
PHQ-9: 9-item Patient Health Questionnaire
T-ICBT: transdiagnostic internet-delivered cognitive behavioral therapy
